# Identification and Validation of a Novel RNA-Binding Protein-Related Gene-Based Prognostic Model for Multiple Myeloma

**DOI:** 10.3389/fgene.2021.665173

**Published:** 2021-04-26

**Authors:** Wei Wang, Shi-wen Xu, Xia-yin Zhu, Qun-yi Guo, Min Zhu, Xin-li Mao, Ya-Hong Chen, Shao-wei Li, Wen-da Luo

**Affiliations:** ^1^Taizhou Hospital of Zhejiang Province Affiliated to Wenzhou Medical University, Linhai, China; ^2^Department of Hematology, Taizhou Hospital of Zhejiang Province Affiliated to Wenzhou Medical University, Linhai, China; ^3^Key Laboratory of Minimally Invasive Techniques & Rapid Rehabilitation of Digestive System Tumor of Zhejiang Province, Taizhou, China; ^4^Department of Gastroenterology, Taizhou Hospital of Zhejiang Province Affiliated to Wenzhou Medical University, Linhai, China; ^5^Health Management Center, Taizhou Hospital of Zhejiang Province Affiliated to Wenzhou Medical University, Linhai, China

**Keywords:** RBP, prediction, prognosis, multiple myeloma, model

## Abstract

**Background:**

Multiple myeloma (MM) is a malignant hematopoietic disease that is usually incurable. RNA-binding proteins (RBPs) are involved in the development of many tumors, but their prognostic significance has not been systematically described in MM. Here, we developed a prognostic signature based on eight RBP-related genes to distinguish MM cohorts with different prognoses.

**Method:**

After screening the differentially expressed RBPs, univariate Cox regression was performed to evaluate the prognostic relevance of each gene using The Cancer Genome Atlas (TCGA)-Multiple Myeloma Research Foundation (MMRF) dataset. Lasso and stepwise Cox regressions were used to establish a risk prediction model through the training set, and they were validated in three Gene Expression Omnibus (GEO) datasets. We developed a signature based on eight RBP-related genes, which could classify MM patients into high- and low-score groups. The predictive ability was evaluated using bioinformatics methods. Gene ontology (GO), Kyoto Encyclopedia of Genes and Genomes (KEGG) enrichment, and gene set enrichment analyses were performed to identify potentially significant biological processes (BPs) in MM.

**Result:**

The prognostic signature performed well in the TCGA-MMRF dataset. The signature includes eight hub genes: *HNRNPC*, *RPLP2*, *SNRPB*, *EXOSC8*, *RARS2*, *MRPS31*, *ZC3H6*, and *DROSHA*. Kaplan–Meier survival curves showed that the prognosis of the risk status showed significant differences. A nomogram was constructed with age; *B2M*, *LDH*, and *ALB* levels; and risk status as prognostic parameters. Receiver operating characteristic (ROC) curve, C-index, calibration analysis, and decision curve analysis (DCA) showed that the risk module and nomogram performed well in 1, 3, 5, and 7-year overall survival (OS). Functional analysis suggested that the spliceosome pathway may be a major pathway by which RBPs are involved in myeloma development. Moreover, our signature can improve on the R-International Staging System (ISS)/ISS scoring system (especially for stage II), which may have guiding significance for the future.

**Conclusion:**

We constructed and verified the 8-RBP signature, which can effectively predict the prognosis of myeloma patients, and suggested that RBPs are promising biomarkers for MM.

## Introduction

Multiple myeloma (MM) is a malignant clonal plasma cell disease of the bone marrow. The main clinical manifestations are monoclonal proteins in the blood or urine and related organ dysfunction (Palumbo and Anderson, 2011). Improved understanding of myeloma and the application of new treatment methods and drugs have greatly improved the survival of patients with myeloma. However, MM is a highly heterogeneous disease, both in response to treatment and in survival, for which the overall survival (OS) of patients ranges from less than 2 years to more than 10 years (Palumbo and Anderson, 2011; Sonneveld et al., 2016). This stark difference may be related to the heterogeneity of myeloma cell biology and multiple host factors (Greipp et al., 2005). Therefore, it is essential to identify disease-related biomarkers and use them to distinguish patients with different prognoses, which will be beneficial for formulating individualized treatments to cope with tumor heterogeneity, thereby improving patients’ final prognosis.

Post-transcriptional gene regulation (PTGR) is a crucial biological process (BP). It is involved in maintaining cellular metabolism, coordinating the maturation, transport, stability, and degradation of all classes of RNAs (Gerstberger et al., 2014). RNA-binding proteins (RBPs) are involved in nearly all steps of PTGR, determining the fate and function of each transcript in the cell, and ensuring cellular homeostasis (Pereira et al., 2017). Gerstberger et al. identified 1542 RBP-associated genes, accounting for 7.5% of all protein-coding genes in humans, and half of these genes are involved in mRNA metabolic pathways. Eleven percent of the RBPs constitute ribosomal proteins, and the rest are involved in multiple non-coding RNA metabolic processes (Gerstberger et al., 2014). RBPs constitute a complex network with cancer-associated RNA targets, and these interactions maintain tumor growth, allowing them to escape death and become more invasive (Tu et al., 2015; Pereira et al., 2017). Overexpression of the *LIN28* paralog was shown to synergize with the Wnt pathway to promote aggressive intestinal adenocarcinoma development in mouse models; it has also been detected in a variety of other solid tumors and hematological malignancies. *LIN28*/*LIN28B* blocks let-7 microRNA (miRNA) biogenesis and, in turn, downregulates the expression of let-7 miRNA target genes, which play an important role in tumor progression and metastasis.

The International Staging System (ISS) distinguishes myeloma patients into stages I, II, and III by serum β_2_ microglobulin and albumin (Greipp et al., 2005). However, this staging only considers the biochemical factors. The R-ISS staging groups patients into stages I, II, and III based on ISS staging, which integrates chromosomal abnormalities (CA) and serum lactate dehydrogenase (LDH) (Palumbo et al., 2015). Although R-ISS distinguishes patients with a good prognosis (stage I) from those with a poor prognosis (stage III), this staging classifies the larger cohort patients into stage II, which is composed of those who still show significant survival heterogeneity (Gonsalves et al., 2020). RBP-associated genes such as *DIS3* have been shown to be associated with myeloma prognosis (Boyle et al., 2020). Here, we identified several prognostically relevant differentially expressed genes (DEGs) for RBP by analyzing public databases and found that these molecular biomarkers can enrich the understanding of myeloma. We also performed Cox regression to construct an 8-gene prognostic model and nomogram that could effectively predict the survival of MM patients and found that this model could improve on the ISS and R-ISS staging ability.

## Materials and Methods

### Data Processing and DEG Identification

All analyses in this study were conducted using R version 4.03. A list of 1542 RBP-related genes was obtained from a previous study (Gerstberger et al., 2014). Gene expression profiles GSE47552, GSE136337, GSE24080, and GSE57317 were downloaded from the Gene Expression Omnibus (GEO) database^1^. The data for MMRF-CoMMpass were obtained from The Cancer Genome Atlas (TCGA^2^). The array data of GSE47552 were obtained using the GPL6244 platform (HuGene-1.0-st Affymetrix Human Gene 1.0 ST Array). GSE136337 was obtained using the GPL27143 platform (HG-U133 Plus 2) Affymetrix Human Genome U133 Plus 2.0 Array; GSE24080 and GSE57317 were obtained using the GPL570 platform (HG-U133 Plus 2) Affymetrix Human Genome U133 Plus 2.0 Array. The data of GSE47552 included bone marrow samples from five healthy donors and 41 newly diagnosed patients with MM. DEGs between MM patients and healthy donors were identified using the R package “limma.” Genes with *P* < 0.05, and [log_2_FoldChange (log_2_FC)] > 1 were considered as DEGs. Volcanic maps and heat maps were drawn using the R package “ggplot2” and “pheatmap” to visualize DEGs.

The Cancer Genome Atlas-MMRF was used as a training set to develop a prognostic signature, while GSE136337, GSE24080, and GSE57317 were used for validation. To meet the needs of this analysis, we set the following conditions to control data quality: (1) Samples must have complete survival information, including survival status and OS time, where death had to be tumor-related and OS time had to be greater than 30 days (2). Samples must have complete R-ISS or ISS information. Finally, 709 cases of MMRF, 559 cases of GSE24080, 559 cases of GSE136337, and 55 cases of GSE57317 were selected for subsequent analysis.

### Gene Ontology and KEGG Enrichment Analysis of DEGs

Gene ontology (GO) term analysis and Kyoto Encyclopedia of Genes and Genomes (KEGG) pathway analysis were performed using the R package “clusterProfiler” to identify the functional roles of the upregulated and downregulated DEGs, respectively. GO enrichment was described from three sub-ontologies: BP, molecular function (MF), and cellular component (CC).

### Gene Set Enrichment Analysis

Gene Set Enrichment Analysis (GSEA) version 4.1.0 was used to explore significant BPs between patients in different risk groups. KEGG gene sets as Gene Symbols^3^ were chosen as the gene set database and the cut-off values for the significance of outcomes were FDR < 0.25, NOM *P* < 0.05, and | NES| > 1.

### RNA-Binding Protein-Related Gene Signature Construction

#### Screening for Hub Genes in the Training Dataset

The TCGA dataset was used as the training cohort, and three datasets (GSE136337, GSE24080, and GSE57317) were used for validation. Univariate Cox regression analysis and multivariate regression analysis (Cox, 1972) were chosen to screen for RBP-related genes that were closely related to the OS of patients. In the univariate Cox regression analysis, *P* < 0.05 was the criterion to screen candidate genes. Next, the least absolute shrinkage and selection operator (Lasso) (Friedman et al., 2010) regression model was applied to minimize overfitting and identify the most significant survival-associated DEGs of RBP-related genes in myeloma. Stepwise multivariate Cox regression analysis was then applied to further establish the RBP-related risk signature. Finally, the hazard ratios (HRs) and regression coefficients of every gene were calculated, and the satisfactory ones were chosen.

#### Construction of the Gene-Related Prognostic Signature in the Training Dataset

The prognostic risk-score signature for prognosis prediction of MM patients was to multiply the expression level of each selected prognostic gene by its corresponding relative regression coefficient weight as follows:

$\mathrm{Risk}\,\mathrm{score}=\sum_{i=1}^{N}\beta \,i\times E\,i$: (*N* represents the total number of signature genes, and βi and Ei represent the coefficient index and the gene expression value of each gene, respectively).

The risk score of each patient and the median risk score were calculated using the training dataset. Those with a higher risk score than the median were classified into the high-score group, while those with a lower risk score were classified into the low-score group. Kaplan–Meier survival curves (Ranstam and Cook, 2017) and receiver operating characteristic (ROC) curves (Kamarudin et al., 2017) of the two groups were plotted to evaluate the sensitivity and specificity of the signature we established.

#### Validation of the Gene-Related Prognostic Signature’s Efficacy in the Validation Datasets

As in the training set, the patients in the validation datasets were classified into the high- and low-score groups by comparing the risk score of each patient with the calculated median risk score from each dataset. The time-dependent prognostic values of the gene signature were investigated using the Kaplan–Meier curve and log-rank test (Kleinbaum, 1998) was used to compare the survival difference between the above-mentioned high- and low-score groups.

### Construction of the Nomogram

In the GSE24080 dataset, we used the lasso regression analysis to analyze all clinical factors and finally selected the clinical prognostic factors together with risk status as the prognostic parameters, ensuring that the nomogram model will not overfit. Then, through “rms” and “regplot” R packages, a prognostic nomogram was established to evaluate the probability of OS in MM patients at 1/3/5/7 years with the regression coefficients based on the lasso analysis. Calibration plots were used to evaluate the discriminative ability of the nomogram. Harrell’s concordance index (C-index) was used to verify the nomogram performance. The ROC curve and calibration curve varying with time were also drawn to estimate the accuracy of the actual observed rate with the predicted survival for 1/3/5/7-year OS of the nomogram. In addition, the clinical application prospects of the eight-gene prognostic signature were determined through decision curve analysis (DCA) (Vickers and Elkin, 2006).

## Results

### Identification of DEGs

**FIGURE 1:**
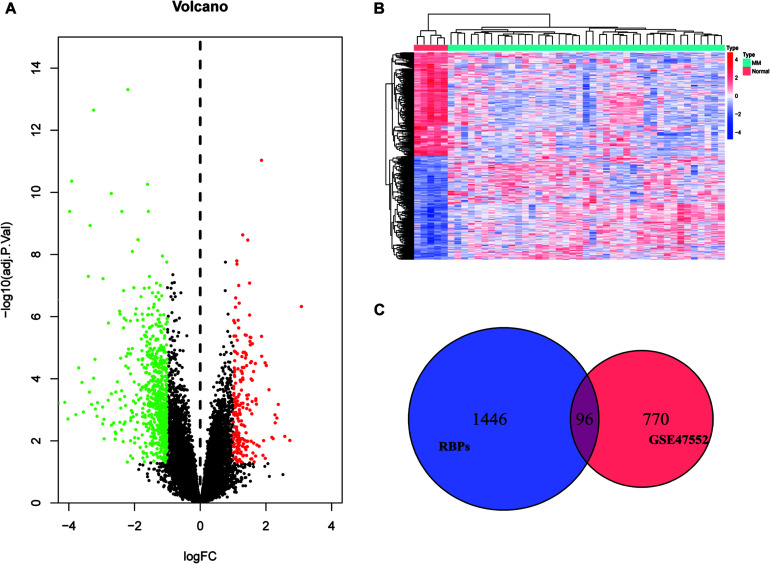
Identification of differentially expressed genes. **(A)** Volcano plot of DEGs; **(B)** heat map of the top 200 DEGs; and **(C)** differentially expressed RBPs.

We set a *P* < 0.05, and [log_2_FoldChange (log_2_FC)] > 1 as the cut-off criterion. Based on this standard, we identified 866 DEGs in MM cases compared with healthy donors, among which 202 were considered significantly upregulated, and 664 were considered significantly downregulated. The volcano plot of DEGs and the heat map of the top 200 DEGs are shown in Figures 1A,B. As shown in Figure 1C, we obtained 96 differentially expressed RBPs by taking the intersection of DEGs and 1,542 RBPs.

### Functional Analysis of Differential RBP Genes

**FIGURE 2:**
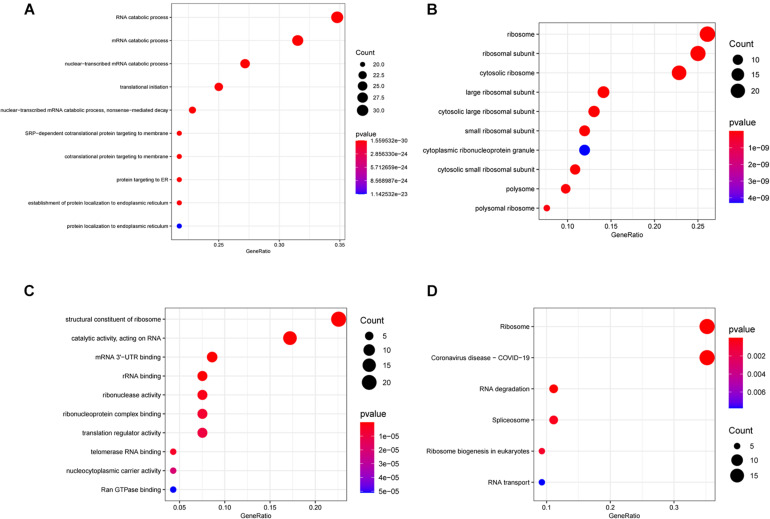
Functional enrichment analysis of DEGs showed by bubble plots. **(A–C)** Three sub ontologies of GO enrichment analysis. **(A)** The biological process (BP) enrichment analysis. **(B)** The cellular component (CC) enrichment analysis. **(C)** The molecular function (MF) enrichment analysis. **(D)** The KEGG enrichment analysis.

For exploring the potential function of these differentially expressed RBPs, we performed GO and KEGG enrichment analysis using the R package “clusterProfiler.” The results of the GO enrichment analysis are presented in three parts. For BP, differentially expressed RNA-binding proteins (DERBPs) were significantly associated with the following terms: RNA catabolic process, mRNA catabolic process, nuclear-transcribed mRNA catabolic process, translational initiation, nuclear-transcribed mRNA catabolic process, nonsense-mediated decay, other important BPs, SRP-dependent cotranslational protein targeting to membrane, cotranslational protein targeting to membrane, protein targeting to ER, the establishment of protein localization to the endoplasmic reticulum, and protein localization to the endoplasmic reticulum (Figure 2A). Four of the top five BP terms were related to various RNA catabolic meaning that these processes may be involved with MM disease progression. The CCs analysis indicated that DERBPs were mostly involved in the following terms: ribosome, ribosomal subunit, cytosolic ribosome, large ribosomal subunit, cytosolic large ribosomal subunit, small ribosomal subunit, cytoplasmic ribonucleoprotein granule, cytosolic small ribosomal subunit, polysome, and the polysomal ribosome (Figure 2B). MF terms were mainly enriched for the structural constituent of ribosome, catalytic activity (acting on RNA), mRNA 3’-UTR binding, rRNA binding, ribonuclease activity, ribonucleoprotein complex binding, translation regulator activity, telomerase RNA binding, nucleocytoplasmic carrier activity, and Ran GTPase binding (Figure 2C). The ribosome, coronavirus disease – COVID-19, RNA degradation, spliceosome, ribosome biogenesis in eukaryotes, and RNA transport pathway terms were significantly enriched in DERBPs, as shown by KEGG enrichment analysis (Figure 2D).

### Exploration of the Prognostic RBPs in MM

**FIGURE 3:**
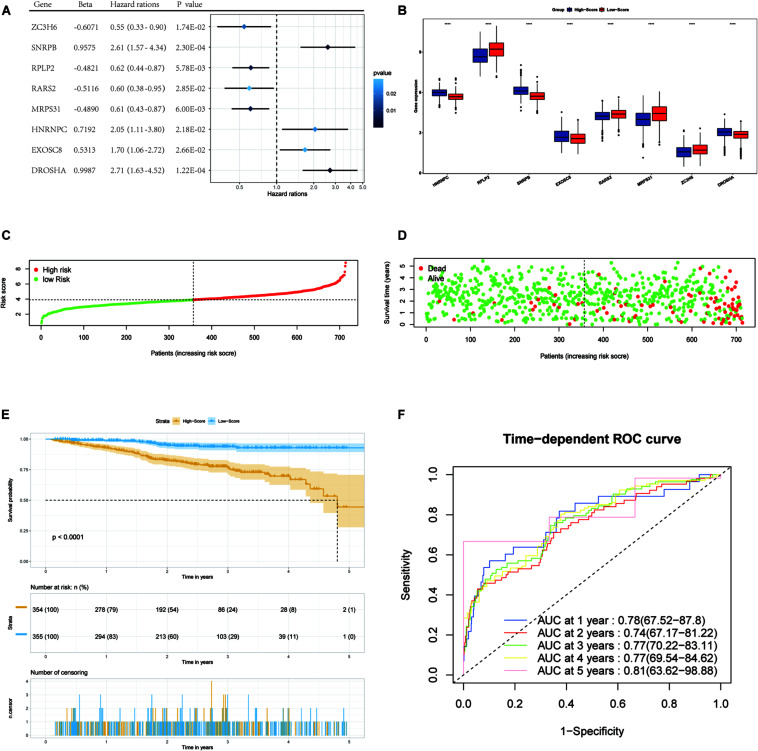
Forest plots of the multivariate Cox regression analysis, the boxplot of eight RBP expression levels, the distribution of risk score, the living status, Kaplan–Meier analysis, and ROC analysis of the eight-gene signature of MM patients in the TCGA cohort. **(A)** Forest plot of the multivariate Cox regression analysis of OS of eight genes. Beta values represent the coefficient index β for each gene. **(B)** The boxplot of eight RBP expression levels in the training set (blue: low-score group. Red: high-score group). **(C)** The distribution of risk scores in the TCGA training set. **(D)** The living status of MM patients in the TCGA training set. **(E)** Kaplan–Meier survival analysis of the low-score and high-score group patients. **(F)** ROC curve analysis according to the 1-to-5-year survival of the area under the AUC value in the training TGCA cohort.

We enrolled 709 patients with a follow-up time of more than 30 days from TCGA as the training dataset for the construction of the signature. Although 96 differentially expressed RBPs were screened before (Figure 1C), only 94 of them were included in the TCGA dataset. The prognostic significance of the 94 genes was investigated using univariate Cox regression. As a result, 34 prognostic-associated candidate RBPs were obtained (*P* < 0.05) (Table 1). LASSO regression was then performed to identify 34 candidate genes closely related to the prognosis of MM patients, including the following 19 genes: *HNRNPC*, *RPLP2*, *SNRPB*, *SNRPE*, *SF3B3*, *KPNB1*, *GAPDH*, *RPS12*, *NFX1*, *MTIF3*, *CIRBP*, *EXOSC8*, *RARS2*, *MRPS31*, *ZC3H6*, *DROSHA*, *NAT10*, *LSM5*, and *PRIM*1 (Supplementary Figure 1). To further screen out the RBPs with the greatest prognostic value, a multiple stepwise Cox regression was conducted to investigate their impact, and eight hub RBPs, *HNRNPC*, *RPLP2*, *SNRPB*, *EXOSC8*, *RARS2*, *MRPS31*, *ZC3H6*, and *DROSHA* were selected to construct the risk model in MM patients (Figure 3A). All of the above genes showed an independent prognostic effect (*P* < 0.05). Among them, *HNRNPC*, *SNRPB*, *EXOSC8*, and *DROSHA* may be regarded as oncogenes, whereas *RPLP2*, *RARS2*, *MRPS31*, and *ZC3H6* may be tumor suppressor genes. The coefficients of these genes indicated their impact on survival prediction.

**TABLE 1:** Unicox results of differential RNA-binding proteins.

Gene	Hazard ratios	CI95	*P-*value
*SUPT4H1*	1.76	1.02–3.06	0.043
*HNRNPC*	3.67	2.1–6.43	0
*RPLP2*	0.66	0.5–0.89	0.007
*SNRPB*	5.09	3.39–7.64	0
*EIF3K*	0.62	0.43–0.9	0.012
*GEMIN5*	1.95	1.31–2.89	0.001
*SNRPE*	2.54	1.79–3.61	0
*UTP6*	2.7	1.59–4.61	0
*SF3B3*	2.24	1.39–3.62	0.001
*KPNB1*	3.97	2.46–6.39	0
*GAPDH*	2.14	1.51–3.03	0
*CNOT1*	1.65	1.06–2.58	0.027
*DDX17*	0.77	0.6–0.98	0.033
*NFX1*	0.54	0.35–0.83	0.005
*MTIF3*	0.52	0.38–0.72	0
*CPSF2*	1.75	1.09–2.81	0.02
*NOL11*	1.88	1.19–2.98	0.007
*ESF1*	2.57	1.57–4.2	0
*CIRBP*	0.5	0.35–0.72	0
*EXOSC8*	1.9	1.28–2.84	0.002
*DDX21*	1.89	1.31–2.73	0.001
*INTS2*	2	1.32–3.01	0.001
*RARS2*	0.58	0.38–0.88	0.01
*MRPS31*	0.59	0.46–0.77	0
*ZC3H6*	0.45	0.29–0.71	0.001
*RPF2*	2.08	1.45–2.99	0
*DROSHA*	2.16	1.3–3.6	0.003
*NAT10*	1.84	1.18–2.89	0.007
*XPO1*	2.14	1.38–3.3	0.001
*LSM5*	1.7	1.05–2.74	0.032
*PRIM1*	2.66	1.97–3.58	0
*CPEB2*	0.7	0.52–0.93	0.014
*SLIRP*	1.69	1.04–2.77	0.035
*DARS2*	1.78	1.35–2.33	0

*CI95: 95% confidence interval.*

### Construction and Validation of the RBP Prognostic Signature

We used the eight hub RBPs selected by multiple Cox regression to establish the eight-gene predictive signature in the TCGA dataset. The risk score for each patient was calculated based on the expression level and the corresponding beta value using the following formula:

Risk score = (−0.6071) × Exp*ZC3H6* + (0.9575) × Exp*SNRPB* + (−0.4821) × Exp*RPLP2* + (−0.5116) × Exp*RARS2* + (−0.4890) × Exp*MRPS31* + (0.7192) × Exp*HNRNPC* + (0.5315) × Exp *EXOSC8* + (0.9987) × Exp*DROSHA*

We then divided MM patients into the low-score group (*n* = 355) and high-score group (*n* = 354) based on the median risk score as the cut-off point. The patients’ gene expression levels, status, and survival time are shown in Figures 3B–D. The K-M results showed that the OS rate of patients in the high-score group was significantly lower than that in the low-score group (*P* < 0.001, Figure 3E). In addition, the time-dependent ROC curve showed that the area under the ROC curve (AUC) of this risk score signature at 1, 2, 3, 4, and 5 years were 0.78, 0.74, 0.77, 0.77, and 0.81, respectively (Figure 3F), indicating that this signature has moderate performance.

**FIGURE 4:**
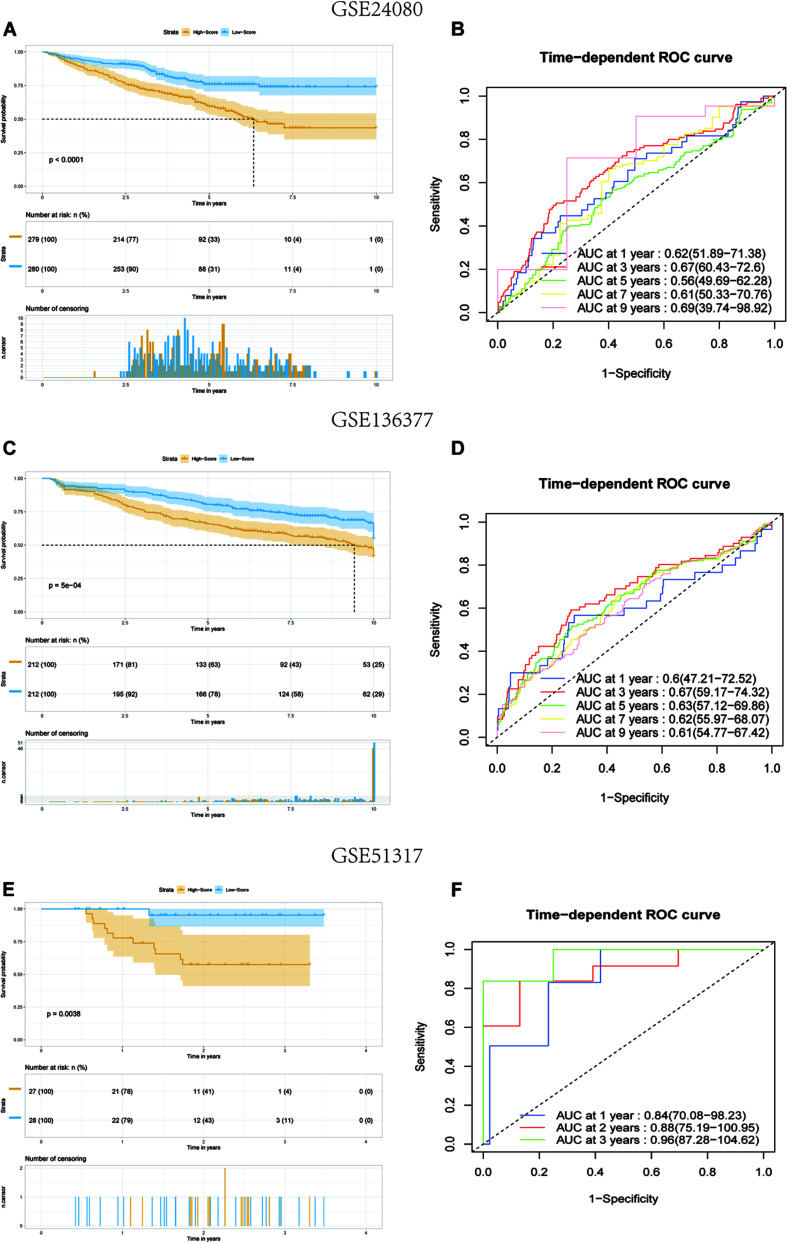
Kaplan–Meier analysis and ROC analysis of 8-gene signature in three validation datasets. **(A,B)** Kaplan–Meier survival analysis of the low-score and high-score group patients and ROC curve analysis according to the 1-, 3-, 5-, 7-, and 9-year survival of the AUC value in the GSE24080 cohort. **(C,D)** Kaplan–Meier survival analysis of the low-score and high-score group patients and ROC curve analysis according to the 1-, 3-, 5-, 7-, and 9-year survival of the area under the AUC value in the GSE136337 cohort. **(E,F)** Kaplan–Meier survival analysis of the low-score and high-score group patients and ROC curve analysis according to the 1-, 2-, and 3-year survival of the AUC value in the GSE51317 cohort.

To verify the predictive value of the 8-gene signature in other MM cohorts, we performed a similar analysis in three datasets: GSE136377, GSE24080, and GSE57317, which all included the risk-related genes selected. The risk score formula described above was validated for the three datasets. We only compared the OS differences of 1–5 years in the TCGA dataset. But when the risk model was applied to the GSE24080 and GSE136337 datasets, comparing for up to 10 years, the results showed that the OS of patients in the high-score group was worse than that of patients in the low-score group (all *P* < 0.01) (Figures 4A,C). The AUC of this risk score signature was >0.6, proving the performance of this scoring system (Figures 4B,D). More interestingly, in the GSE57317 dataset, the OS difference between the two groups was very significant, with AUC values of 0.84,0.88, and 0.96 at 1, 2, and 3 years, respectively, proving the prognostic value in this dataset (Figures 4E,F). In conclusion, this scoring model exhibited acceptable performance for all three datasets.

### Establishment and Validation of Nomogram Survival Model

#### Univariate and Multivariate COX Regression Analysis of the Model

**FIGURE 5:**
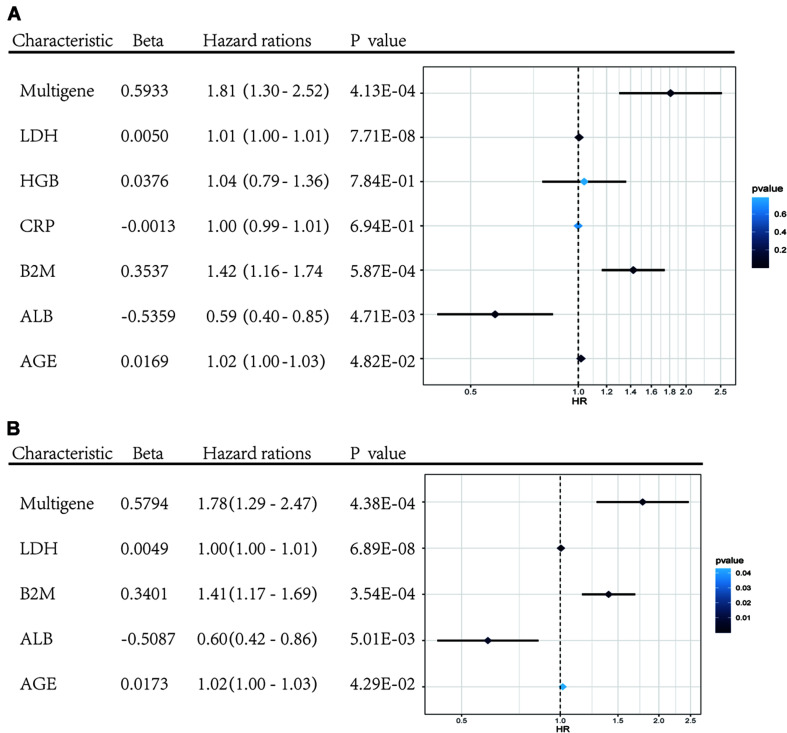
Forest plots of the multivariate and univariate Cox regression analysis in GSE24080 cohorts. **(A)** Forest plot of the univariate Cox regression analysis OS of the clinical factors and risk score. **(B)** Forest plot of the multivariate Cox regression analysis OS of clinical factors screened by univariate Cox analysis and risk score. Beta values represent the coefficient index β for each clinical factor.

Univariate and multivariate Cox regression analyses were performed using clinical data from the GSE24080 dataset. Using univariate Cox regression analysis, age, *B2M*, *CRP*, *LDH*, *ALB*, *HGB*, and risk score status were selected to assess the independent prognostic factors in the MM sample (Figure 5A). Multivariate Cox regression analysis confirmed that age (HR = 1.02, 95% CI [1.00−1.03]; *P* = 0.042), *B2M* (HR = 1.41, 95% CI [1.17−1.69]; *P* = 0.000354), *LDH* (HR = 1.00, 95% CI [1.00−1.01]; *P* = 6.89 × 10^–8^), *ALB* (HR = 0.60, 95% CI [0.42−0.86]; *P* = 0.005), and multigene risk status (HR = 1.78; 95% CI [1.29−2.47]; *P* = 0.000438) were significant independent risk factors (Figure 5B). Based on the results shown in Figure 3C, the risk score can be used as an independent prognostic factor without being affected by clinicopathological features. The HR of the high-risk group was 1.78 (95% CI: 1.29−2.47) times higher than that of the low-risk group.

#### Nomogram Construction

**FIGURE 6:**
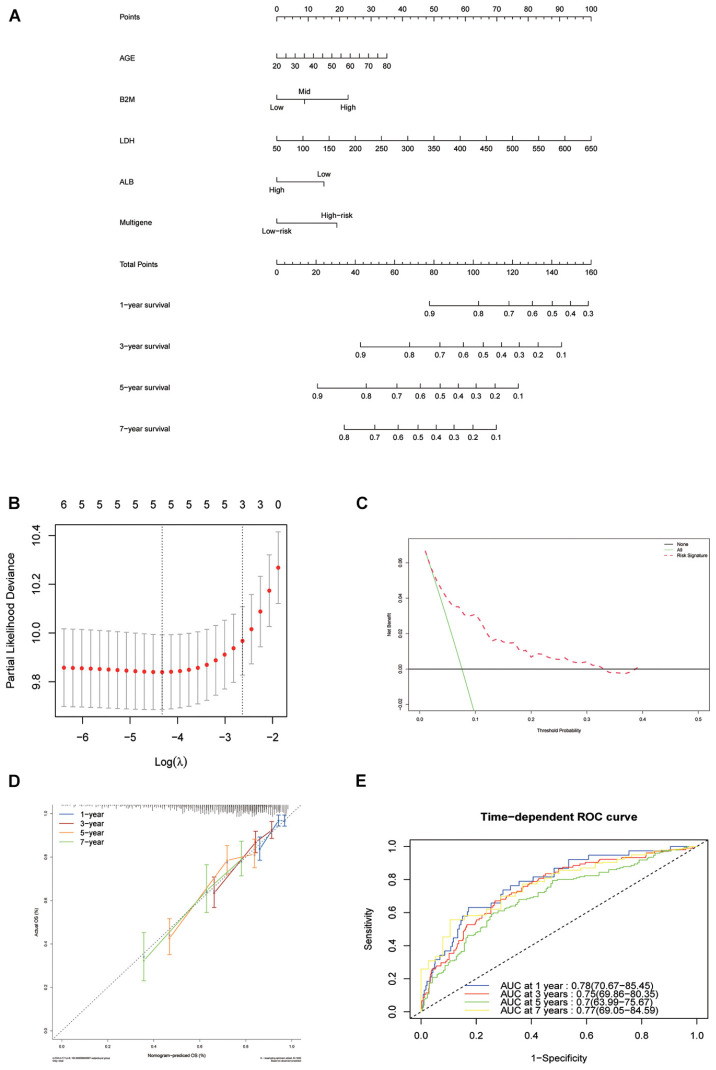
Nomogram construction based on the eight-gene signature and prognostic value of genes. **(A)** The nomogram for predicting the proportion of patients with 1-, 3-, 5-, and 7-year OS of MM. **(B)** LASSO regression analysis used tenfold cross-validation via the maximum criteria. **(C)** Decision curve analysis of nomogram predicting 1-, 3-, 5-, and 7-year OS of MM. **(D)** Calibration plots of the nomogram. **(E)** Time-dependent ROC analysis of nomogram predicting 1-, 3-, 5-, and 7-year OS of MM.

To establish a clinical method to predict the survival probability of MM patients, we created a nomogram using lasso regression analysis to estimate the probability of, 1-, 3-, 5-, and 7-year OS with age, *B2M*, *LDH*, *ALB*, and risk score status (Figures 6A,B). The AUC of 1-, 3-, 5-, and 7-year OS predictions were 0.78, 0.75, 0.70, and 0.77, respectively (Figure 6E). The calibration curve was used to describe the prediction value of the nomogram, and the 45° line indicates the actual survival outcomes. The results for predicting 1-, 3-, 5-, and 7-year OS showed that the nomogram-predicted survival closely matched the best prediction performance (Figure 6D), indicating that the nomogram has a significant predictive value for predicting 1-, 3-, 5-, and 7-year OS in patients with MM. The concordance index (C-index) was calculated to evaluate the prognostic capability of the model. The C-index of the nomogram was 0.71 (95% CI [0.69–0.73]), which proved that the nomogram’s value a good predictive tool for MM prognosis. We used DCA analysis to confirm the range of the threshold probabilities for a prediction model. As shown in Figure 6C, the nomogram threshold probability based on 8-gene combinations was significantly better than the default strategies of treating all or none at a threshold probability > 0.05.

### Validation of Classification Capabilities of the Eight-Genes Prognostic Signature for R-ISS and ISS Stage II Patients

**FIGURE 7:**
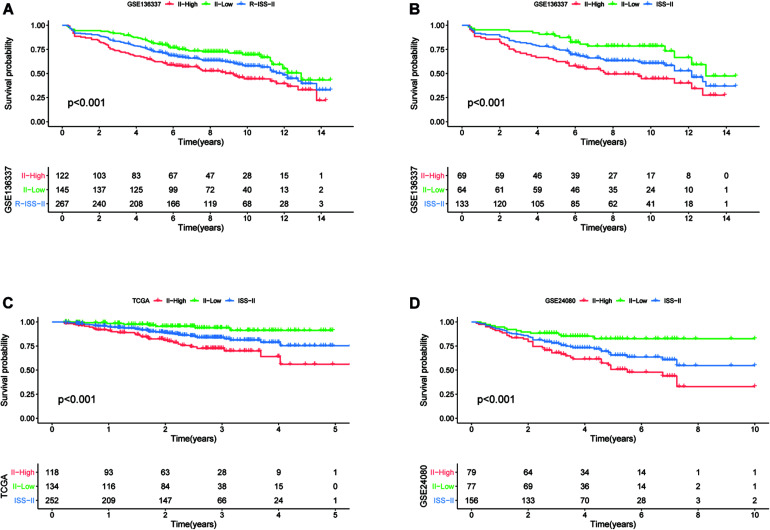
The eight-gene model can enhance the predictive power of R-ISS and ISS for their respective stage II cohorts. **(A)** R-ISS stage II in GSE136337. **(B–D)** ISS stage II in GSE136337, TCGA-MMRF, and GSE24080. (Red: a group that was reclassified as high risk. Green: a group that was reclassified as low risk. Blue: total group before reclassification.)

To assess whether our model could improve the heterogeneity of patients with R-ISS stage II, we reclassified R-ISS stage II patients in GSE136337 based on the model. Finally, 122 of 267 patients were redefined as II-High, while 145 were defined as II-Low, and the survival curves were subsequently plotted. To highlight the discriminatory effect, we defined the categorized patients as R-ISS II co-plotted in graphs. As shown in Figure 7, stage II patients were clearly divided into two groups with different survival, and patients defined as II-High had a worse prognosis. Meanwhile, we found that the classifier also optimized for ISS stage II in GSE136337, and it was validated in two other independent datasets (Figures 7B–D). We treated R-ISS stage I and stage III in the same way, but the discrimination was not ideal (Supplementary Figures 2A,B). To further evaluate whether a similar effect would be apparent on ISS, we performed the same reclassification for the TCGA, GSE24080, and GSE136337 datasets. In GSE136337, the results were not significant for either ISS stage I or stage III (*P* > 0.05). For GSE24080, the difference in survival after grouping was significant only for ISS stage I (*P* = 0.033), but the effect of differentiation was not good enough. Surprisingly, the TCGA dataset performed the best in the three datasets. Although each dataset performed differently, the overall results were not as good as those of stage II (Supplementary Figures 2C–H).

### Signaling Pathways Analysis of High-Risk Group

**FIGURE 8:**
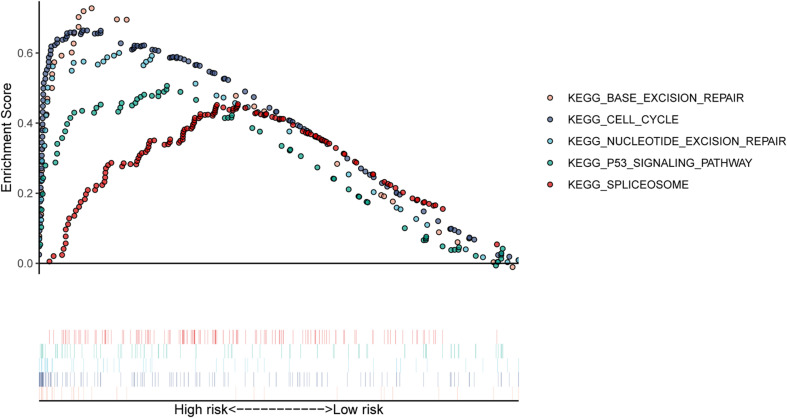
The KEGG pathways were enriched in the high-risk group by performing the GSEA analysis.

In our study, patients in the high-risk group exhibited worse survival. We used GSEA to investigate the potentially important pathways causing different prognoses in the two groups. A KEGG functional enrichment analysis showed that the base excision repair, nucleotide excision repair, spliceosome, cell cycle, and p53 signaling pathways may be involved in cancer development (Figure 8). The spliceosome pathway also appeared in the KEGG enrichment results of DERBPs, further demonstrating the importance of this pathway.

## Discussion

With the development of novel diagnostic approaches and treatment strategies, the survival of patients with MM has improved. However, MM remains an incurable disease for the vast majority of patients (Rajkumar, 2020). To ensure the predictive value of RBP-associated genes, we first screened for RBPs with significant differences in expression between newly diagnosed myeloma patients and normal human bone marrow. Subsequently, an eight-gene prognostic signature was established based on the expression levels of RBP-associated genes. By calculating the risk scores, we divided all patients into high- and low-score groups in the training dataset and three validation datasets, respectively. The predictive ability of this scoring model was evaluated and verified in the training set and the three validation datasets. Meanwhile, we built a nomogram survival model to predict the 1/3/5/7-year survival rate by combining age, *B2M*, *LDH*, *ALB*, and risk score status.

The role of RBPs in promoting cancer has been confirmed, and *DROSHA*, *EXOSC8*, *HNRNPC*, *MRPS31*, *RPLP2*, and *SNRPB* have also been reported to be related to the occurrence and development of a variety of tumors. *DROSHA* and DICER are important factors involved in miRNA processing. For neuroblastoma, the expression level of *DROSHA* decreased in advanced-stage patients and was associated with poor prognosis (Lin et al., 2010). *EXOSC8* is an essential component of the exosome complex and is involved in RNA surveillance and epigenetic regulation. Cui et al. found that the expression of *EXOSC8* in colorectal cancer was higher than that in normal tissues in a public database, indicating a poor prognosis. They confirmed that the expression of *EXOSC8* in colorectal cancer was higher than that in matched normal tissues in clinical samples, and verified the cancer-promoting effect of the gene in cell and animal experiments (Cui et al., 2020). As an RBP, *HNRNPC* was reported to be aberrantly expressed at elevated levels in a variety of tumors, besides being involved in some well-established BPs, such as RNA splicing. Further, it was found to control endogenous dsRNA and downstream interferon response functions and is indispensable to a subset of breast cancer cell lines, and partial suppression of this gene can affect cell line activity (Wu et al., 2018). Xu et al. (2014) found that mitochondrial ribosomal protein S31 (*MRPS31*) was associated with thyroid cancer disease progression using a machine-learning method. Ribosomal P2 (*RPLP2*) is an ancient ribosomal stalk protein. It has been shown that *RPLP2* can alleviate ribosome pausing in the DENV envelope coding sequence, thus enhancing protein stability. This effect is achieved by improving the efficiency of co-translational folding. *RPLP2* also influences multipass transmembrane protein biogenesis, making it important in protein synthesis. Moreover, it is associated with DNA repair, proliferation, apoptosis, and tumorigenesis, and is significantly associated with malignancies such as gynecological tumors, digestive system tumors, and lung adenocarcinoma (Campos et al., 2020). The *SNRPB* of SMB/B’, the core member of the spliceosome mechanism, promotes cell proliferation and inhibits cell apoptosis. Changes in the core splice protein encoded by *SNRPB* may interrupt RNA processing, resulting in specific changes in the splice of variable exons, thus affecting the entire transcription process (Correa et al., 2016). Besides its important role in splicing, *SNRPB* mutations also have significant effects on cell division and DNA repair (Kittler et al., 2004). *SNRPB* is associated with poor prognosis in a variety of cancers, including glioblastoma, non-small cell lung cancer, and metastatic prostate cancer (Yi et al., 2009). Although the above-mentioned genes have been reported in a variety of cancers, their precise roles in myeloma remain unknown; thus, our study may provide direction for further exploration. *RARS2* encodes mitochondrial arginine tRNA synthetase, a protein essential for the translation of all mitochondrially synthesized proteins (Edvardson et al., 2007). Mutation of the *RARS2* gene causes destructive effects on the cerebellum and cerebellum-associated nuclei (inferior olivary nuclei, pontine base, and dentate nuclei), leading to degenerative changes in the brain. However, the exact mechanism of this effect remains to be elucidated (Joseph et al., 2014). *ZC3H6* is a zinc finger transcription factor, but little is known about its function or expression. However, we found that *ZC3H6* may be closely related to the prognosis of patients with MM. This finding has not been mentioned in previous literature, so it may be a potential research direction in the future. In previous studies, *RARS2* and *ZC3H6* have not been reported to be associated with tumors. In our study, these two genes were differentially expressed in myeloma and correlated with patient survival, suggesting that these two genes are potential tumor-related genes that require further investigation. The vast majority of these eight RBP genes were first reported to be associated with myeloma, and in the future, we intend to establish a real-world cohort of MM patients to validate the value of these genes again.

To explore how RBPs are involved in the development and progression of MM, we performed GO and KEGG enrichment analyses of 96 DERBPs. In the GO enrichment analysis section, the results of enrichment from BP, CC, and MF are described. The BP results show that the RNA catabolic process was the most significantly enriched result. The CC results suggest that RBPs are mainly localized in the ribosome and its associated locations. MF then reflects the involvement of RBPs in the structural conformation of the ribosome, RNA catalytic activity, and other important MPs. KEGG indicated that RBPs affected the disease by participating in the ribosome, RNA degradation, spliceosome, and RNA transport pathways. The results of conducting enrichment analysis only on DEGs may miss the contribution of genes that are relevant but less biologically significant to disease, so we further analyzed the differences in BPs between high-risk and low-risk groups by GSEA. In the GSEA-KEGG results, as with the results of the KEGG enrichment analysis of the DERBPs, the “Spliceosome pathway” was suggested to be significant. The RNA splicing pathway is associated with a variety of human tumors (Wang and Aifantis, 2020). In MM, aberrant RNA splicing patterns were found to exist, and patients with a large number of novel splice loci tended to have worse survival outcomes, which could be used to distinguish extremely high-risk groups (Bauer et al., 2020). These findings fit our enrichment results, demonstrating the value of the spliceosome pathway in myeloma, but there are currently few relevant studies, and its role in myeloma remains to be comprehensively uncovered. One study showed that spliceosome interference was an unreported mechanism of action of proteasome inhibitors; inhibition of the spliceosome could synergize with carfilzomib to potentiate antitumor effects, suggesting that targeted spliceosome therapy could serve as a future research direction for the treatment of myeloma (Huang et al., 2020).

R-ISS staging had the advantage of distinguishing patients with a very good prognosis (stage I) from those with a very poor prognosis (stage III); however, more patients were classified as stage II. Although stage II patients were intermediate in terms of overall prognosis, the issue of significant heterogeneity within stage II patients has not been addressed. In this study, we constructed a model that we intended to be a powerful predictor of patient survival. Therefore, we wanted to evaluate whether the model could enhance R-ISS prediction. The results showed that the model could further discriminate patients with R-ISS stage II, but performed poorly in stage I and III patients. This result not only further suggests intra-patient heterogeneity at stage II, but also illustrates that our model can optimize R-ISS to some extent. Besides this, we also applied this model for ISS staging in the three databases. The results were similar between the three databases, the optimization effect of the model on the ISS stage II phase was the most obvious, and it had a smaller optimization effect on stages I and III, although the effect was weaker than R-ISS. The distinct results for stages I and III affirm the ability of R-ISS to discriminate between patients with stages I and III diseases, as well as the significant heterogeneity within patients with stage II disease, while also demonstrating the ability of our model to optimize both R-ISS and ISS.

Collectively, we suggest that our 8-RBP-related gene signature and nomogram could be practical and reliable prognostic tools for MM. Although the signature and nomogram showed excellent performance in the training and validation sets, they inevitably had some limitations. First, although it performed well in predicting the survival of patients with MM, it lacked verification of large-scale prospective trials. Second, the R-ISS data were only obtained from the GSE136337 database, and further confirmation is needed to conclude that our model can enhance the predictive power of R-ISS. Third, the associated mechanisms have not been validated in MM cells. Based on this, our follow-up research will focus on verifying the conclusions of this study in terms of clinical applications and molecular mechanisms.

In conclusion, we introduced a prognostic signature based on eight RBP genes that might be independent prognostic factors in MM and a novel nomogram that could predict the survival of patients with MM.

## Data Availability Statement

The original contributions presented in the study are included in the article/Supplementary Material, further inquiries can be directed to the corresponding authors.

## Author Contributions

WW, SX, XZ, QG, and MZ participated in the design of the study and performed the statistical analysis. XM, YC, SL, and WL drafted the manuscript. All authors read and approved the final manuscript.

## Conflict of Interest

The authors declare that the research was conducted in the absence of any commercial or financial relationships that could be construed as a potential conflict of interest.

## Footnotes

1 https://www.ncbi.nlm.nih.gov/geo/

2 https://tcga-data.nci.nih.gov/

3 http://www.gsea-msigdb.org/gsea/downloads.jsp

## Supplementary Material

Supplementary Figure 1 Lasso cox regression of the MM-related genes in the TCGA dataset.

Supplementary Figure 2 The optimization ability of the 8-gene model for stages I and III is not as good as that for stage II (either R-ISS or ISS). **(A,B)** R-ISS in GSE136337. **(A)** R-ISS stage I; **(B)** R-ISS stage III. **(C–H)** ISS in GSE24080, GSE136337, and TCGA-MMRF. **(C)** ISS stage I in GSE24080. **(D)** ISS stage III in GSE24080. **(E)** ISS stage I in GSE136337. **(F)** ISS stage III in GSE136337. **(G)** ISS stage I in TCGA-MMRF. **(H)** ISS stage III in TCGA-MMRF. (Red: a group that was reclassified as high-risk. Blue: a group that was reclassified as low risk.)
