# Supplementary figures and images for: Identification and Validation of a Novel RNA-Binding Protein-Related Gene-Based Prognostic Model for Multiple Myeloma
Source: Front Genet. 2021 Apr 26;12:665173. doi: 10.3389/fgene.2021.665173 (PMC8107400; doi:10.3389/fgene.2021.665173)

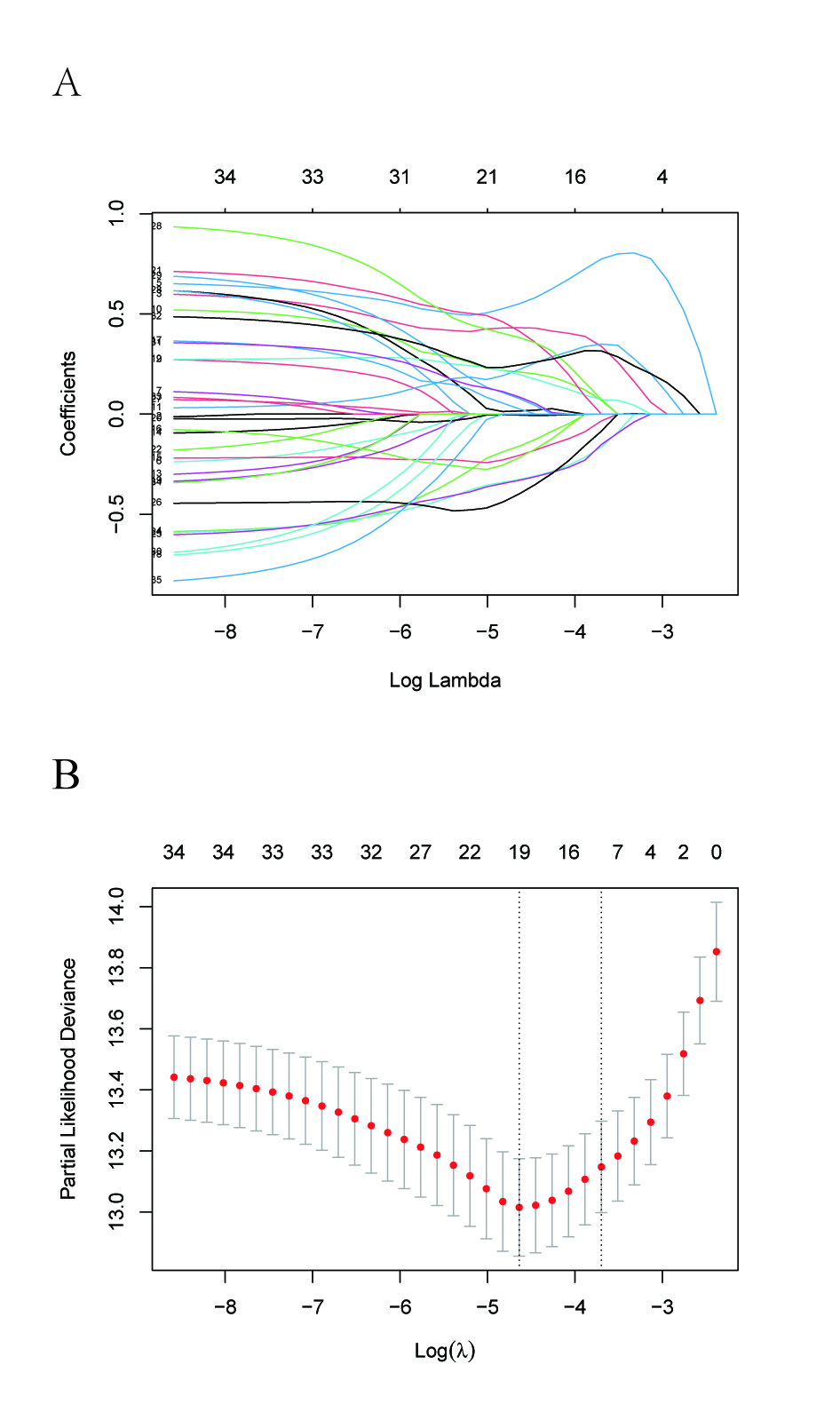

Supplement: Supplementary Figure 1 — Lasso cox regression of the MM-related genes in the TCGA dataset. [file Image_1.TIF]

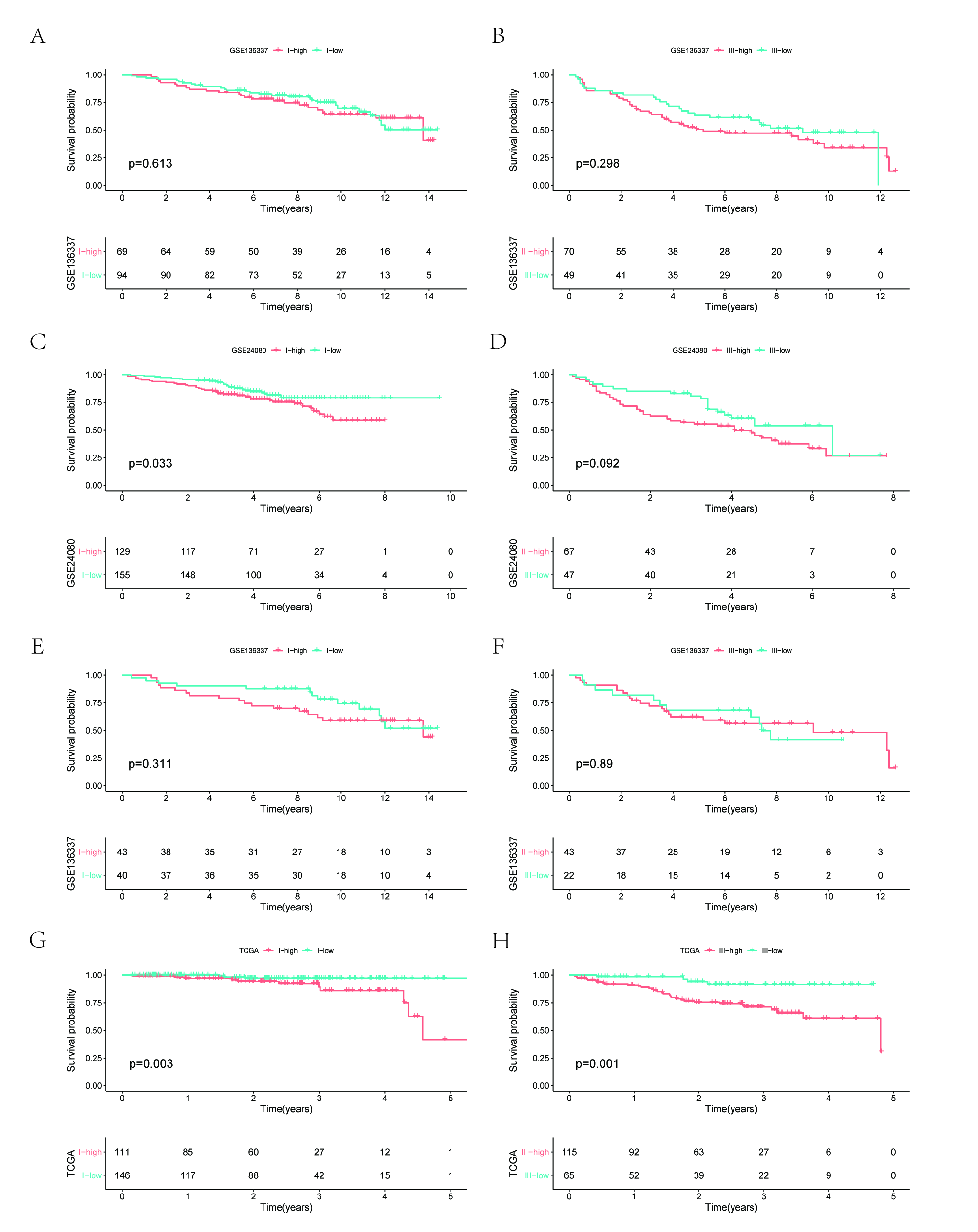

Supplement: Supplementary Figure 2 — The optimization ability of the 8-gene model for stages I and III is not as good as that for stage II (either R-ISS or ISS). (A,B) R-ISS in GSE136337. (A) R-ISS stage I; (B) R-ISS stage III. (C–H) ISS in GSE24080, GSE136337, and TCGA-MMRF. (C) ISS stage I in GSE24080. (D) ISS stage III in GSE24080. (E) ISS stage I in GSE136337. (F) ISS stage III in GSE136337. (G) ISS stage I in TCGA-MMRF. (H) ISS stage III in TCGA-MMRF. (Red: a group that was reclassified as high-risk. Blue: a group that was reclassified as low risk.) [file Image_2.TIF]
